# LATENT HEALTH PROFILES ASSOCIATED WITH INSTITUTIONAL ADMISSION AFTER SNF STAY AMONG MEDICARE PLWH

**DOI:** 10.1093/geroni/igad104.1348

**Published:** 2023-12-21

**Authors:** Brianne Olivieri-Mui, Sandra Shi, Ellen McCarthy, Gahee Oh, Dae Hyun Kim

**Affiliations:** Northeastern University, Boston, Massachusetts, United States; Hebrew Senior Life, Roslindale, Massachusetts, United States; Hebrew Senior Life, Roslindale, Massachusetts, United States; Hebrew Senior Life, Roslindale, Massachusetts, United States; Hebrew SeniorLife, Roslindale, Massachusetts, United States

## Abstract

**Background:**

Health profiles may help identify PLWH at risk for institutional admission after a short-term skilled nursing facility (SNF) stay.

**Methods:**

We estimated rates of institutional admission in one-year follow-up after discharge from a SNF stay (<100 days) among Medicare FFS beneficiaries with HIV (2014-2019). Latent profile analysis identified subgroups based on prevalent conditions one year prior to discharge including 8 indices: mental health (MH range 0-6), substance use (SU 0-4), cardiovascular conditions (CV 0-9), sensory (SE 0-4), musculoskeletal (MU 0-10), pulmonary (PU 0-2), learning disabilities (LD 0-9), and other chronic conditions (OCC 0-11). Cox models estimated the association between latent profiles and time to institution admission (hospital, nursing home), adjusting for demographics, dual eligibility, HIV treatment; censoring included death and study end.

**Results:**

The 618 PLWH studied were male (73%), with mean age 60 (standard deviation [sd] 11). Two latent health profiles were: CV+OCC+MH (n=77), median values CV=3, OCC=2, MH=1; healthy (n=541), median 0 for all. Compared to the healthy group, the CV+OCC+MH group had similar days spent in an institute (60 days [96] vs 66 days [96]), and similar hazards for institutional admission in adjusted models (HR:1.09, 95% CI [0.81-1.46]) in one-year after SNF discharge. HIV treatment for ≥80% of the time prior to discharge had lower hazards (HR:0.76 [0.59-0.98]) and dual eligibility had higher hazards (HR:1.32 [1.09, 1.61]) of returning to an institutional setting.

**Conclusions:**

Latent profiles had similar risk for institutional admission in one-year after discharge from a SNF stay, despite having significantly different contributing factors.

